# Pathways to help-seeking for sexual difficulties in older adults: qualitative findings from the third National Survey of Sexual Attitudes and Lifestyles (Natsal-3)

**DOI:** 10.1093/ageing/afaa281

**Published:** 2021-01-28

**Authors:** Sharron Hinchliff, Ruth Lewis, Kaye Wellings, Jessica Datta, Kirstin Mitchell

**Affiliations:** University of Sheffield, Division of Nursing and Midwifery, Sheffield S10 2LA, UK; University of Glasgow, MRC/CSO Social & Public Health Sciences Unit, Glasgow, UK; London School of Hygiene and Tropical Medicine, Faculty of Public Health, Environments and Society, London, UK; London School of Hygiene and Tropical Medicine, Faculty of Public Health, Environments and Society, London, UK; University of Glasgow, MRC/CSO Social & Public Health Sciences Unit, Glasgow, UK

**Keywords:** help-seeking, sexual difficulties, older adults, qualitative, Natsal

## Abstract

**Background:**

Older adults are at an increased risk of sexual difficulties due to ageing and chronic health conditions. While they experience barriers to seeking and receiving help for sexual difficulties there is a dearth of research about the help-seeking journey.

**Objective:**

To explore decision-making in context; particularly, the reasons why older adults do, or do not, seek help for sexual difficulties.

**Methods:**

Semi-structured interviews were conducted with 11 men and 12 women aged 58–75 who reported having a health condition, disability or medication that had affected their sex life in the last year. Participants were part of the third British National Survey of Sexual Attitudes and Lifestyles (Natsal-3). Data were analysed thematically.

**Results:**

Help-seeking was rarely a predictable or linear process. Participants tended to wait and see if the sexual difficulty got better on its own or improved as a result of lifestyle changes. An often-lengthy period of thinking, researching and planning could end with a decision to seek professional help, to not seek help, or do nothing for now. A significant barrier was concern about the interaction of medicines prescribed for the sexual difficulty with those already taken for chronic health conditions. Patient fear of not being taken seriously and doctor reticence to ask thwarted potential conversations. Help-seeking journeys often ended without resolution, even when professional help was sought.

**Conclusions:**

To give patients and practitioners permission to raise the topic, suggestions include providing patients with a pre-consultation card which lists topics they would like to talk about, including sexual issues.

## Key Points

- Sexual activity and intimacy are key components of quality of life for older adults.
- But older adults are at an increased risk of experiencing sexual difficulties due to ageing and chronic health conditions.
- Help-seeking for a sexual difficulty is not a predictable or linear process.
- Older adults tend to think, do their own research, and plan before seeking professional help or deciding not to.
- Many help-seeking journeys end without resolution.

## Background

Sexual activity and intimacy are important across the adult life-course, and many older adults view both as quality of life components with benefits to health and well-being [1]. However, with increasing age comes the likelihood of experiencing a sexual difficulty and research indicates that older adults face barriers to seeking and receiving help [2]. To date, little is known about the decision-making process of older adults when considering seeking help for sexual difficulties.

Sexual difficulties are common in middle and older age [3]. The most recent National Survey of Sexual Attitudes and Lifestyles (Natsal-3) (of which this paper is part) found that just under a third of men aged 65–74 had erection difficulties, over a third of women lacked interest in sex, and one in five women experienced uncomfortable vaginal dryness [4]. An earlier survey of adults aged 40–80 found that the most common sexual problems for sexually active men in the UK were early ejaculation (20%) and erectile dysfunction (18%). For sexually active UK women, it was lack of interest (33.7%), lack of pleasure (25.2%) and inability to orgasm (23.6%) [5]. Sexual difficulties are a ‘less visible’ aspect of chronic health conditions: currently in the UK, around 15 million people have at least one chronic condition [6]. Living with a chronic condition can affect sexual enjoyment in a number of ways. For example, sexual difficulties can be the result of diseases such as diabetes which can harden and narrow the blood vessels and cause erectile problems and reduced vaginal sensitivity. Conditions which are painful, such as arthritis, affect a person’s mobility or comfort when having sex.

Sexual difficulties can be caused by the medicines prescribed to manage chronic health conditions. Common drugs with side-effects which may affect sexual activity include cardiovascular drugs, anti-epileptics, and cancer drugs such as long-acting gonadotrophin-releasing hormone agonists [7]. Individuals with multi-morbidities take numerous medications, and older adults have an increased sensitivity to medicines which can make the treatment of iatrogenic sexual dysfunction challenging. Less obvious difficulties arise from taking medication, for example dry mouth [8], which can interfere with sexual intimacy. Additional ways in which chronic conditions can impact sexual activity and pleasure include the psychological adjustment to diagnosis and to body-altering treatments, which can affect sexual self-esteem [9]. Chronic illness may mean a shift in roles for those in relationships as one partner becomes dependent on the other, which can reduce sexual attraction and desire [10]. And those with an illness that affects sexual function may lack the confidence to meet new partners [11].

Research has found that older adults with sexual difficulties are at an increased risk of depression [12]. Qualitative research conducted as part of the English Longitudinal Study on Ageing identified a connection between sexual difficulties and feelings of frustration, sadness, and relationship breakdown [13]. Distress plays a role in decision-making, and a higher degree of distress about a sexual difficulty increases the likelihood of discussing it with a physician [13–15]. Though a wide range of staff, including geriatricians, treat medical conditions that can affect patients’ sexual lives, a recent cross-European survey with participants aged 60–75 found that the first stop for those with a sexual difficulty was their general practitioner (GP)/primary-care physician, and the main reasons for seeking help were that ‘sex was important to me’ and ‘sex was important to my relationship’ [15]. Not all older adults with a sexual difficulty seek professional help: reasons include waiting for it to go away, assuming it is a normal part of ageing, embarrassment and fear of an ageist response from the doctor [15, 16].

Practitioners report barriers to discussing sexual well-being with older patients, including time constraints, lack of training, embarrassment, and concern about invading patient privacy [16]. There is a paradox in that older patients want doctors to ask them about sexual problems whereas doctors want patients to raise the question themselves [17, 18]. Consequently, there is unmet patient need when the practitioner does not ask and the patient does not tell.

In contrast to the extensive research on sexual difficulties in older age which takes a biomedical focus, the research on help-seeking is scarce. We know little about how older adults negotiate the psychological and social factors that inform their decision to seek help. Such findings would be useful for practitioners to develop resources and shape the services they provide. In this qualitative study, we address a gap in the literature by exploring decision-making in context, particularly the reasons why older adults do, or do not, seek help for sexual difficulties.

## Methods

The third National Survey of Sexual Attitudes and Lifestyles (Natsal-3) is a probability sample survey of women and men aged 16–74 living in private households in Britain. Overall, 15,162 adults completed the survey, of whom 3,343 were aged between 55 and 74. The response rate for Natsal-3 was 57.7% and the co-operation rate was 65.8%. Fieldwork took place between 2010 and 2012. The full survey methodology and ethical approval are reported elsewhere [19].

For the present study, in-depth interviews were conducted with a sub-sample of participants aged 55–74 who took part in the survey. Eligible participants were the 388 men and 281 women who reported having a health condition, disability, or medication that had affected their sex life in the last year. We asked survey participants whether they would be willing to be contacted again about a subsequent interview: 80% agreed. We drew a sample guided by recency of Natsal-3 participation, gender distribution, and geographical spread across Britain. Letters of invitation for the interview were sent, which the researcher followed up by phone to reiterate the purpose of the interview and ask about willingness to take part. Face-to-face interviews were conducted with 11 men and 12 women in their homes. Participants gave signed consent including to digitally record the interview, and were provided with an information sheet and a list of agencies from which they could seek advice on topics raised, if required. The sex, age, and relationship status of participants, plus their experiences of sexual difficulties and help-seeking, are shown in Table 1.

**Table 1:** Characteristics of participants and their experiences of help-seeking

**Participant**>	**Gender**	**Age**	**Relationship status**	**Experience of sexual difficulty and help-seeking**
M1	Male	75^a^	Married (second)	Takes Erection-enhancing medicine (EEM), wife does not know. Help-seeking not discussed.
M2	Male	61	Married (second)	Takes EEM, sought help for ED but angina puts him off using EEM (fear of heart attack), tried sex aides for wife and a plunger for ED
M3	Male	69	Married (40 years)	Tried EEM but did not like the lack of spontaneity. Sought help from GP
M4	Male	62	Married (41 years)	Tried EEM in the past, sought help twice and thinking of seeking help again
M5	Male	58	Single (gay)	No sexual desire, help-seeking not discussed
M6	Male	63	Separated	Wife saw doctor at his request about her lack of desire, came off medication and her desire returned
M7	Male	64	Married (8 years)	EEM, help seeking not discussed
M8	Male	64	Married	Has ED, considered seeking help but has not due to embarrassment
M9	Male	62	Widowed (previously married 30 years)	Tried EEM but does not need it
M10	Male	70	Single	EEM offered at hospital, he accepted it but has not used it as he has no sexual desire
M11	Male	71	Married (49 years)	ED, sought help but the doctor was unhelpful
W1	Female	71	Married (long-term)	Not sexually active, no sexual desire, has not sought help
W2	Female	69	Married (45 years)	Not sexually active, might seek help from nurse
W3	Female	67	Separated (previously married 40 years)	Not sexually active
W4	Female	71	Married (53 years)	No sexual desire but has sex for husbands pleasure to ‘stop him going elsewhere’
W5	Female	69	Married (third time)	Not sexually active, no sexual desire, husband has ED, have not sought help
W6	Female	59	Cohabiting (long-term)	No sexual desire, she and partner do not miss sex
W7	Female	68	Single	Not sexually active, not sought help as it is not an issue
W8	Female	74	Married (50 years)	Sexual difficulties due to pain, has sought help in the past
W9	Female	64	Married (46 years)	Husband will not have sex and will not seek help
W10	Female	65	Married (33 years)	Has a long-term illness but no health practitioners have mentioned sex to her at all
W11	Female	59	Widow (was married 27 years)	She received advice on sex after hysterectomy
W12	Female	59	Married (43 years)	Husband has sought help for ED

^a^This participant turned 75 in between the survey and the interview.

Interviews explored health status and sexual activity, whether health issues affected sexual activity and pleasure, the relationship context, and action taken by participants in response to health-related sexual difficulties. We undertook a thematic analysis drawing on the principles of Grounded Theory [20] where a close reading of the transcripts, followed by open-coding and constant comparison of the data, enabled the identification of key themes. KM coded the entire dataset in NVivo. The theme on experiences of sexual difficulties, and stories of seeking help (or otherwise), was interrogated for this article by SH who undertook further coding to identify subthemes. The analysis was refined with the aid of text searches in NVivo, identification of ‘deviant’ cases, and continual back and forth reading between raw data and the emerging synthesis of the data. A separate article from Natsal-3 explored sexual activity and sexual satisfaction in this group [11]; here, we focus on help-seeking.

## Results

**Figure 1:**
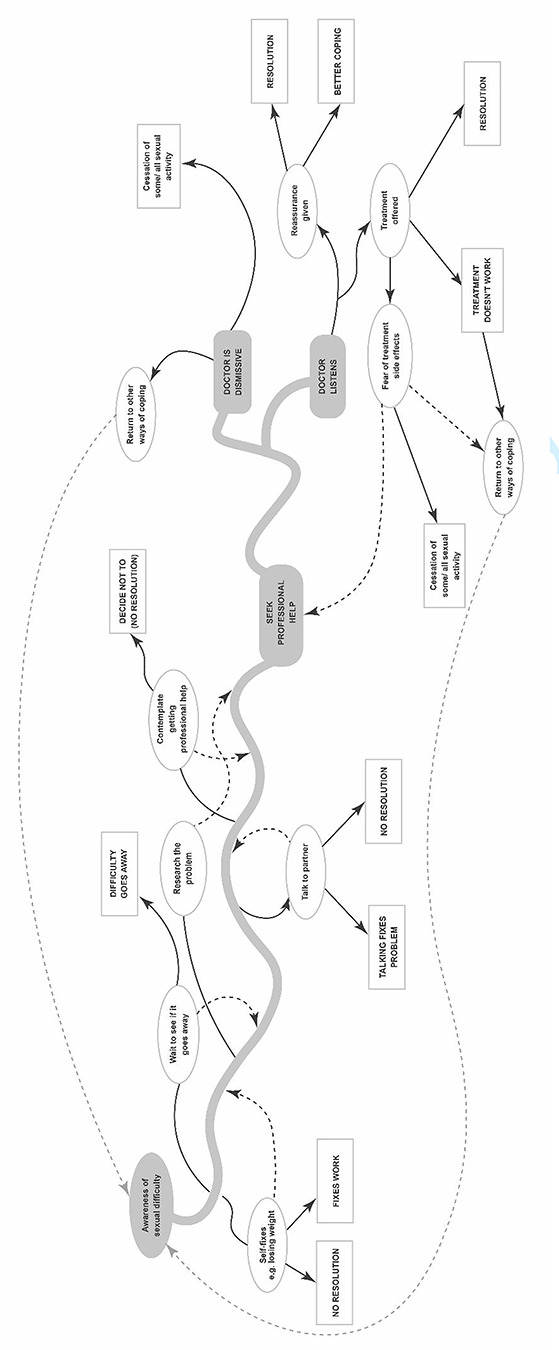
The help-seeking pathway.

The majority of participants were married, currently or most recently in different-sex relationships, and aged in their 60s. All had a health condition or took medication that affected their sex life: the most common issue reported by men was erectile difficulties (ED), and women was low/no sexual desire. However, both women and men largely framed help-seeking in relation to ED and erection enhancing medicines (EEMs). We explore these issues through the analogy of a journey (Figure 1), which analysis revealed was a useful framework for understanding help-seeking.

### The long and winding road: pathways to help-seeking

Seeking professional help for a sexual difficulty was rarely described as a predictable or linear process. Rather than seek help at the first signs of a sexual difficulty, participants tended to wait and see if it got better on its own, or improved as a result of their own ‘fixes’ which included lifestyle changes or trying different sexual positions.

To be absolutely honest I would feel a little bit of a fraud because I do feel that if I am going to speak to the doctor about that [ED], the least I should do is lose weight because that’d probably have an effect… I would just feel, going to the doctors and saying about possibly Viagra, I think ‘I should be doing other things first’ (Man aged 64)

For this participant, making lifestyle changes to tackle the health behaviours which he believed contributed to his sexual difficulty caused a delay in seeking professional help. We do not know, however, if he was reluctant to seek help and used the need to lose weight as an excuse to postpone help-seeking. This participant took hypertensive medication for high blood pressure but seemed unaware of the drug’s potential sexual side-effects.

Participants spent a long time, sometimes years, thinking about whether or not to seek professional help. For many, this entailed carrying out their own research by searching for information online. The desire to identify the cause of a sexual difficulty formed a strong motivating factor for seeking help and tended to follow failed attempts to self-identify the cause. For example, one participant sought help from his GP after deciding that his alcohol use did not contribute to his ED. He undertook ‘lots of reading and research’ on the Internet first as a process of elimination.

I don’t know whether it was my high blood pressure or the tablets from it, but increasingly from the time I was 50 I suffered from my erection not being firm enough… especially if I’d had a lot of alcohol, and then sometimes it was hard enough for [partner] to enjoy, sometimes it wasn’t, and quite often I didn’t orgasm… You start off thinking ‘It’s only drink’, then it starts to happen when you don’t have drink (laughs)… I’ve blamed different things at different times. (Man aged 62)

In contrast to the man in the earlier extract, this participant knew about the sexual side-effects of his medication. His concern over his partner’s sexual pleasure was a driver to seeking help. Although this was not commonly expressed by men, it challenges the traditional heterosexual narrative that men prioritise their own sexual pleasure. The situation, however, is complex:

Over the years I’ve been very frustrated… when I started blaming cystitis it, in a sense, killed my sex life but it didn’t stop it because I thought ‘I can do this for [husband]’. (Woman aged 74)

In the absence of sexual desire or presence of sexual pain, engaging in intercourse was an attempt by this woman to find a meaningful solution when help-seeking had failed. Sexual pleasure was described in relation to the importance of sex within the relationship, and thus influenced whether a sexual difficulty caused distress and required action.

Clearly, motivation to overcome the difficulty was an important factor in setting out on the help-seeking journey. But for some, not talking about the sexual issue was linked to not seeking help. A reluctance, or inability, to talk about sex with a partner closed down opportunities to explore avenues of help. Feeling embarrassed to talk about sexual difficulties was implicit throughout the data. Examples include the privacy of sex (‘My private life is very private’: Man aged 69) and that talk among friends, even partners, was regarded as taboo. Some women tried to talk about a sexual issue with their husband/partner but maintained that he refused to speak about it.

He is 73 and I don’t think he’d admit it but he’s not really up to it, and really I couldn’t be bothered… he doesn’t talk about things like that, you couldn’t even broach that subject because that would be to undermine his manliness. (Woman aged 69)

Not being able to get an erection was perceived by this participant to be damaging to her husband’s self-esteem. She appears to have lost interest in sex herself, which demonstrates why it is important to view sexual issues in the context of the relationship.

Thinking, researching, and planning constituted the first steps in the help-seeking journey, which ended with a decision to seek professional help, not to seek help, or do nothing for now. Indeed, some participants believed that help-seeking was only appropriate up to a certain age, beyond which it was ‘too late’. They accepted the discontinuation of their sex life and rationalised it as something that ‘just happens’ in older age. For them, the sexual difficulty did not negatively affect their relationship. For others, dealing with health conditions could relegate sexual issues as a lesser area of concern.

### Destination or pit stop? Beliefs about resolution and physician response

Help-seeking could lead to the resolution of the problem and the end of the journey. More commonly, it was simply a waymark, heralding the next journey stage and decisions to be made (Figure 1). Fear of the potential side-effects of treatment was raised by men in relation to EEMs, and with reference to the other prescribed medicines they were already taking.

After I had the heart trouble, then the ‘shall I, shan’t I, what effect is this going have on me, is it going to give me a heart attack?’ And that’s one of the things that has led to the actual downward slope of our sex life, because I’m a bit frightened of taking it… I asked the doctor… he didn’t give me an answer, not like straight yes or no, he hedged his bets ‘Oh yeah, you should be all right’. (Man aged 61)

Deciding whether to use sexual medicines involved weighing-up the pros and cons, and considering whether to prioritise sexual pleasure above perceived risk to health. While the participant had previously used EEMs, he did not get the reassurance he sought from his GP about safety given his current health status. This led to a discontinuation of EEMs and the cessation of his sex life.

Several participants were concerned that their sexual difficulty would not be taken seriously by the doctor. In particular, there was a fear that GPs would not consider ED to be a medical difficulty unless it resulted from a health condition (e.g. prostate disease). This was sometimes borne out of experience.

It seems to be - they can’t give you the cure unless you pay for it, and they don’t consider it to be a true medical difficulty. (Man aged 61)

Doctors did not always view sexual difficulties as ‘legitimate’ health difficulties. In this way, general health was prioritised over sexual health. Some participants viewed their sexual difficulty as more pressing than their health condition which also influenced the help-seeking journey. But sometimes, the response of the doctor could make participants feel that they were not being taken seriously.

I went to the doctors and they said ‘Well, you’ve got to want to [have sex]’, I said ‘I want to but nothing’s happening’… That was in my late 50s, and I went again probably a couple of years after to see a different doctor and it was exactly the same response… After a while I was just not bothered. I think the wife was getting frustrated because I couldn’t and, of course, that impacted her moods… [it] changed the appetite. Yes, because the more I felt that I couldn’t, the worse it was. (Man aged 71)

An outcome of not receiving appropriate care was strain on the relationship and an end to his sex life. The anticipation of not being able to get an erection appeared to make it more difficult to achieve an erection.

Having access to an approachable doctor served to facilitate help-seeking by shortening the delay and smoothing the journey. An example below is from a participant who specifically mentioned ‘the better doctor’ who he planned to consult about his sexual difficulties. Indeed, some participants described compassionate doctors and helpful consultations which reassured and supported them.

I went for advice from her and said ‘Is this a condition that can be sorted out?’ I mentioned I’d read about various aids that were available and we had a long chat and she was very sympathetic and said that this happens to countless numbers of people, you’re not unique, don’t worry about it. (Man aged 69)

Similarly, one female participant questioned if she was the cause of her husband’s ED. But the ‘very, very, good doctor’ reassured her otherwise.

[The doctor said] It is to do with his medication and he was on some different medication for a while and that made him very depressed, and this is the side-effect of something else. So I’d rather him be happy and loving than have the sex and, well we wouldn’t have had the sex in any case, been too bloody miserable to do it. (Woman aged 59)

While some doctors listened and took the problem seriously, this was after the topic had been raised by the participant. In the main, participants were not asked about their sexual well-being by doctors even though all participants had health conditions which are known to interfere with sexual life.

## Discussion

Capturing the voices of older adults has provided new insights into the help-seeking journey for those with sexual difficulties, and added to the limited evidence base in this area. Seeking help was not a straightforward or linear process. For example, participants tended not to seek help when they first experienced a sexual difficulty: they made lifestyle changes, waited to see if the problem got better on its own, and did their own research, mainly online, to identify the cause and cure (Figure 1). The decision-making process involved the consideration of the importance of sexual activity within the relationship alongside potential risks such as the safety of taking medication. Indeed, a significant barrier, which to our knowledge has not been reported empirically before, was concern about the possible interaction of taking a prescribed medicine for the sexual difficulty in addition to those already taken for chronic health conditions. This is an important point given that people are living for longer, and models that project disease burden have identified that multi-morbidity will grow significantly over the next 20 years [21].

Some participants in the present study never embarked on a help-seeking journey, while others began (i.e. they considered their options) but never reached a destination that brought a resolution. It could be that there was less urgency, especially for participants in long-term relationships, to get their difficulties resolved because they felt they had more time available. When participants sought help, this was chiefly because of the importance of sex within the relationship and the impact of the sexual difficulty on sexual pleasure. This finding resonates with other studies which have identified that older adults who seek help for a sexual difficulty do so because of the distress they and their partners experience [13, 16, 22]. This finding supports the English Longitudinal Study on Ageing but provides more depth as those data were collected during a survey where only limited textual responses could be given [13]. Another reason for seeking help was the desire to know the cause of their sexual difficulty. This contrasts with earlier research which identified fear of the underlying cause as a barrier to help-seeking for sexual difficulties in the older population [23], and demonstrates diversity in the reasons why older adults do or do not seek help. Embarrassment and viewing sexual difficulties as ‘normal’ with ageing were also part of the journey: both support the findings of earlier studies [5, 23, 24].

Help-seeking was influenced by whether or not participants could talk about a sexual difficulty with their partner. There was evidence that avoiding such discussions was linked to the view that ED impaired a sense of masculinity. This resonates with studies which have found that a common response for men with sexual difficulties is to remain silent [25, 26]. However, some men in the present study did seek help, which again shows diversity in men’s experiences. For example, the Men’s Attitudes to Life Events and Sexuality study [27] examined masculinity, quality of life and ED in men aged 20–75 and found that being in ‘good health’ and having a ‘good relationship with wife/partner’ were significant components of quality of life, more so than ‘having a satisfying sex life’. Men with ED reported lower personal satisfaction in all quality of life components, and in particular with regard to satisfaction with sex life, but they found no differences in constructs of masculinity between men with ED who sought treatment and those with ED who did not.

An interesting point is that participants in the present study talked about their sexual issues with the interviewer, yet some felt unable to talk to their partner or a health practitioner. This contrast highlights the unique context of the interpersonal relationship, in that talking about sex with a stranger (albeit a professional researcher in a confidential interview) can be easier than talking about it with a close friend or intimate partner.

Clearly, the help-seeking journey was not linear and had many pit stops prior to reaching the destination. This information can help health practitioners to better support patients. While previous studies have identified that the GP is the main source of professional help for older adults with sexual difficulties, many health practitioners including geriatricians are involved in their care. Implications for healthcare practice include practitioner awareness that when a patient has sought help, there is likely to have been a delay, and they have built-up courage to raise the problem. Also, that their partner may have prompted the help-seeking.

Sexual difficulties should thus be treated with sensitivity. King *et al.* [28] found a discrepancy between professional and patient understandings of what constituted a sexual difficulty, therefore, it is important to seek to understand what the patient views as problematic rather than being solely directed by diagnostic criteria. We recommend that practitioners reassure patients that sexual pleasure can be obtained through a varied sexual repertoire and that what counts as sex may not require an erect penis, which may help to dispel notions of ED as a threat to masculinity. Practical suggestions for health practitioners include giving patients a pre-consultation card which lists topics (including sex) they would like to talk about. This gives both the patient and practitioner permission to raise the topic: an issue identified as important to sexual communication in this population [29]. Practitioners could use the card to guide the consultation, thus addressing the earlier point where patients want doctors to ask them about sexual matters.

This study adds to the evidence base on older adults’ sexual difficulties by introducing new insights into the help-seeking journey, in particular concerns about drug interactions. A strength is that participants were recruited from the British National Survey of Sexual Attitudes and Lifestyles which includes those who had, and had not, sought help, thus going beyond studies where participants are recruited only from clinics after seeking help. It is possible that taking part in an interview appealed to men with erection difficulties because Viagra has made it more acceptable to talk about erection difficulties [30]. While this reduces gender bias in the present sample, it indicates that more research is required to explore gender differences and the help-seeking journey. Interestingly, women were more likely than men to talk about the effect of their partner’s health condition on their sex lives. These gendered nuances require further exploration through qualitative research. To extend the evidence base, research is also required with populations who were not represented in this study (e.g. those who do not identify as heterosexual).

## Conclusion

Sexual well-being in the context of multi-morbidities is an important area of healthcare, particularly regarding patient concerns about the effects of taking medication. Sexual activity and intimacy are increasingly recognised as key components of healthy ageing. Given that the global population is ageing and that sexual difficulties can have a significant negative impact on an individual’s quality of life, it is imperative that we support older adults to seek help if they wish to.
